# Enhanced Photocatalytic Removal of Cyanotoxins by Al-Doped ZnO Nanoparticles with Visible-LED Irradiation

**DOI:** 10.3390/toxins13010066

**Published:** 2021-01-17

**Authors:** Majdi Benamara, Elvira Gómez, Ramzi Dhahri, Albert Serrà

**Affiliations:** 1 Laboratory of Physics of Materials and Nanomaterials Applied at Environment (LaPhyMNE), Faculty of Sciences, University of Gabes, Erriadh Manara Zrig, 6072 Gabes, Tunisia; majdibenamara1@gmail.com; 2 Thin Films and Nanostructures Electrodeposition Group (GE-CPN), Department of Materials Science and Physical Chemistry, University of Barcelona, Martí i Franquès 1, E-08028 Barcelona, Spain; e.gomez@ub.edu; 3 Institute of Nanoscience and Nanotechnology (IN2UB), University of Barcelona, E-08028 Barcelona, Spain; 4 Department of Engineering, University of Messina, 98166 Messina, Italy; 5 ITE Sarl Laboratories for Analysis Water, Processing and Packaging Industry (ITE), Moukondo, Brazzaville, Congo

**Keywords:** cyanotoxins, microcystin-LR, anatoxin-(a), photocatalysis, ZnO-doped nanoparticles, visible light, LEDs

## Abstract

The ZnO-based visible-LED photocatalytic degradation and mineralization of two typical cyanotoxins, microcystin-LR (MC-LR), and anatoxin-A were examined. Al-doped ZnO nanoparticle photocatalysts, in Al:Zn ratios between 0 and 5 at.%, were prepared via sol-gel method and exhaustively characterized by X-ray diffraction, transmission electron microscopy, UV-vis diffuse reflectance spectroscopy, photoluminescence spectroscopy, and nitrogen adsorption-desorption isotherms. With both cyanotoxins, increasing the Al content enhances the degradation kinetics, hence the use of nanoparticles with 5 at.% Al content (A5ZO). The dosage affected both cyanotoxins similarly, and the photocatalytic degradation kinetics improved with photocatalyst concentrations between 0.5 and 1.0 g L^−1^. Nevertheless, the pH study revealed that the chemical state of a species decisively facilitates the mutual interaction of cyanotoxin and photocatalysts. A5ZO nanoparticles achieved better outcomes than other photocatalysts to date, and after 180 min, the mineralization of anatoxin-A was virtually complete in weak alkaline medium, whereas only 45% of MC-LR was in neutral conditions. Moreover, photocatalyst reusability is clear for anatoxin-A, but it is adversely affected for MC-LR.

## 1. Introduction

Cyanobacteria are common prokaryotic photosynthetic inhabitants of bodies of water worldwide that can survive in extreme environmental conditions and through dramatic changes in salinity, temperature, desiccation, and irradiance [1,2]. Under favorable conditions, cyanobacteria can achieve high rates of population density in water and thus facilitate the formation of dense blooms that can significantly compromise water quality (e.g., increase the turbidity of freshwater and change its odor and taste) [3,4,5]. Even so, the greater threat to freshwater resources posed by cyanobacterial blooms is the formation of cyanotoxins, which, in naturally occurring concentrations, are highly toxic to plants, invertebrates, and vertebrates [6,7]. Because the ingestion of cyanotoxins generally causes liver, digestive, and neurological diseases, their presence could make water too dangerous for recreational activities, drinking-water purification, fisheries, and, more broadly, human health [1,2,8,9].

Cyanotoxins are typically classified as hepatotoxins (e.g., microcystin, nodularin, and cylindrospermopsin), neurotoxins (e.g., anatoxin and saxitoxin), or dermatoxins (e.g., aplysiatoxin), depending on their toxicological effects [1,2,8,10]. However, they can also be classified according to their chemical composition—that is, as cyclic peptides (e.g., microcystin and nodularin), alkaloids (e.g., cylindrospermopsin, anatoxin, and saxitoxin), or lipopolysaccharides (e.g., endotoxin). No exhaustive list of cyanotoxins exists, however, and far more could be discovered, pending the identification of cyanobacterial secondary metabolites. Regardless of classification, cyanotoxins generally present a high risk of illness and mortality and rank among the most lethal groups of known biotoxins [8,9,10,11].

In recent years, mounting evidence of increased cyanobacterial blooms and cyanotoxins worldwide has raised public awareness of their toxic effects on water use and water quality. Research has shown that the increased prevalence, occurrence, and abundance of cyanobacterial blooms, and, in turn, concentration of cyanotoxins in bodies of water on a global scale, are driven by eutrophication, rising concentrations of atmospheric CO_2_, and global warming, all of which stem from anthropogenic activities, especially climate change [2,9,11,12]. Because more frequent and prolonged blooms are likely to occur worldwide in the coming decades, the global context of the threat urges the development of simple, low-cost strategies and technologies for detoxicating and degrading cyanotoxins that can be readily implemented worldwide [2,9].

Although conventional water-treatment processes, such as coagulation, flocculation, rapid sand filtration, sedimentation, and chlorination, can efficiently remove cyanobacteria and low levels of various cyanotoxins, they cannot completely degrade some cyanotoxins (e.g., anatoxin-A). In the past two decades, the relatively low efficiency of those methods gave way to the use of advanced oxidation processes (AOPs) [9,13,14,15,16]. In general, AOPs are based on the mineralization of organic pollutants by highly reactive and non-selective species generated in situ (i.e., H_2_O_2_, ·OH, O_2_·^−^, and O_3_). Among the different species generated, hydroxyl radicals are often considered to be the most important contributors to water decontamination during AOPs [17,18]. As a testament to their versatility, AOPs can generate such reactive species by accommodating various processes, especially radiation, oxidation, and catalysis, and even their combination [9,13,14].

Of the various AOPs, heterogeneous photocatalysis, particularly when using TiO_2_-based photocatalysts, has emerged as an efficient approach to degrading, and eventually mineralizing, microcystins and cylindrospermopsin by using UV-light irradiation [9,12,13]. However, despite considerable efforts to improve TiO_2_’s photocatalytic performance in mineralizing cyanotoxins under visible light or sunlight irradiation, usually via doping or surface modification, such laboratory-scale research has rarely assessed the integration of the photocatalysts into photocatalytic reactors [19]. On top of that, few studies have involved investigating the photocatalytic degradation of other cyanotoxins, such as anatoxin-A, or the use of alternative, inexpensive sources of light [9,13,20,21]. Importantly, it is clear that photocatalysis does not seem to be the most appropriate way to remove cyanotoxins from a body of water (e.g., reservoirs, lakes, and ponds), but it may be especially relevant for treating water that contains cyanotoxins (and other organic contaminants), which is destined for human, animal, or industrial use.

Research in the future may face formidable challenges with improving the economic, practical, engineering, and environmental quality of full-scale applications that can promote the use of photocatalysis in water-treatment processes [9]. Whereas solar light, despite its advantages, entails the complexity of designing efficient solar photocatalytic reactors and, beyond that, using solar light, a smarter way to facilitate photocatalytic reactor design for full-scale plants could be by using light-emitting diodes (LEDs) as a light source. LEDs are characterized by low energy consumption, which, in turn, reduces the energy consumption of UV or visible lamps; they can also simplify photoreactor designs [9,13,22].

In our study, a visible-light active Al-doped ZnO nanoparticle photocatalyst, synthesized by following a facile, scalable sol–gel new method, was examined for how the Al:Zn ratio affected its morphology, structure, and optoelectronic properties. The photocatalytic activity of the Al-doped ZnO nanoparticle photocatalyst was evaluated according to its photocatalytic degradation of microcystin-LR (MC-LR) and anatoxin-A, under visible-LED irradiation, compared with the photocatalytic activity of ZnO and Al-doped ZnO nanoparticles. The effects of the dosage of photocatalyst and the solution’s pH were also investigated, as were its reusability and chemical and photochemical stability.

## 2. Results and Discussion

Al-doped ZnO photocatalysts, all synthesized via the sol-gel method, were labeled A0ZO, A1ZO, A3ZO, and A5ZO for Al:Zn atomic ratios of 0%, 1%, 3%, and 5%, respectively. The synthesized materials were characterized by X-ray diffraction (XRD), transmission electron microscopy (TEM), UV-vis diffuse reflectance spectroscopy, photoluminescence (PL) spectroscopy, and nitrogen adsorption-desorption isotherms.

### 2.1. Characterization of Photocatalysts

#### 2.1.1. Microstructural and Morphological Characterization

**Figure 1:**
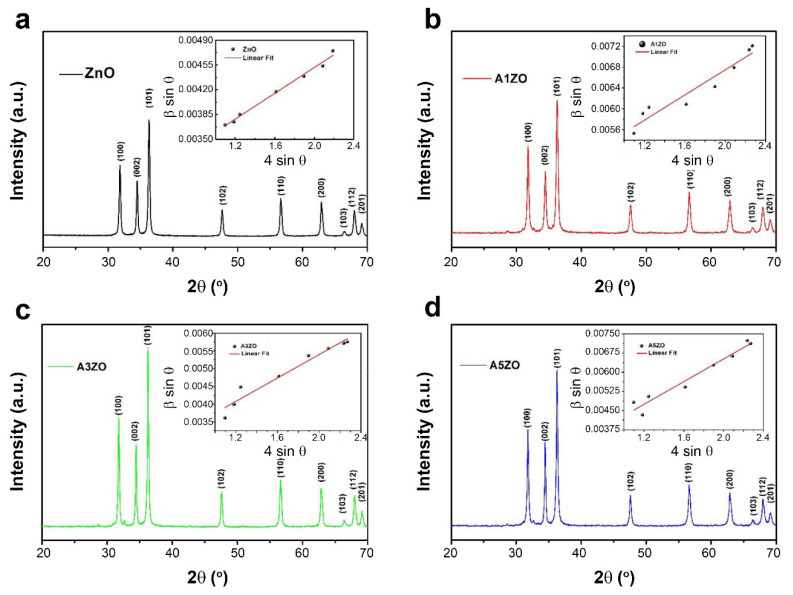
X-ray diffraction patterns of (**a**) A0ZO, (**b**) A1ZO, (**c**) A3ZO, and (**d**) A5ZO nanopowders with Williamson-Hall plots (inset).

The diffractogram in Figure 1 illustrates the XRD patterns of Al-doped ZnO (AZO) nanoparticles annealed at 400 °C and their numerous diffraction peaks. As shown, all peaks had different intensities, which could indicate anisotropic growth. Notably, the peaks corresponded to a reticular plane of (100), (002), (101), (102), (110), (103), (200), (112), and (201) in ZnO’s hexagonal structure, with space group P63mc, according to JCPDS no. 01-073-8765. The calculated lattice parameters values (*a,c*), the concluded unit cell volume (*V*), the average sizes of the crystallites (*D*), the micro-deformations (*ε*), and the dislocation densities (*δ*) are presented in Table 1 [23,24,25,26]. Among other results, the smaller average size (*D*) and higher dislocation density (*δ*) of A1ZO indicate its relatively high concentration of structural defects. Structural defects can improve the photocatalytic performance of ZnO-based photocatalysts.

**Figure 2:**
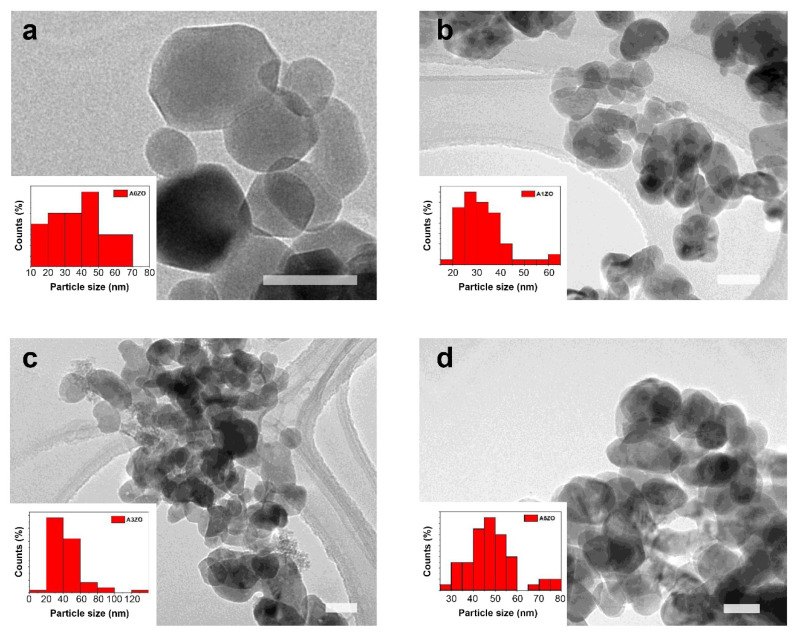
Transmission electron microscopic images and grain sizes distribution of (**a**) A0ZO, (**b**) A1ZO, (**c**) A3ZO, and (**d**) A5ZO nanopowders. Scale bar: 50 nm.

The distribution of the AZO samples prepared by the sol-gel method by shape and particle size appears in multiple TEM images in Figure 2. Pure ZnO nanoparticles were widely distributed in terms of size and irregularly in shape. Doping ZnO with Al, by comparison, produced smaller nanoparticles. Low doping with Al appeared to reduce the size of particles, while higher loadings generated not only more of the small nanoparticles but also nanoparticles greater than 60 nm in size. The small particles were likely derived from the presence of secondary phases formed during heterogeneous nucleation and the accompanying spontaneous increase in the grain area [27]. For comparison’s sake, Table 1 lists the average particle sizes of AZO samples estimated by TEM images. On average, particles in the sample, with low doping (i.e., A1ZO), were smaller than the ones in the pure ZnO samples and the samples with high doping (i.e., A3ZO and A5ZO).

The surface area of pure ZnO and Al-doped ZnO nanopowders was investigated by the BET measurements. The surface areas of pure ZnO was found to be 11.8 m^2^/g. The surface area increased to 16.6, 19.2, and 17.8 m^2^/g when ZnO was doped with 1, 3, and 5 at.% of Al, respectively. The increase in surface area could be accounted for by the change of size of the particles. A similar increase in surface areas was observed in other studies involving doping on ZnO with aluminum.

#### 2.1.2. Optoelectronic Characterization

**Figure 3:**
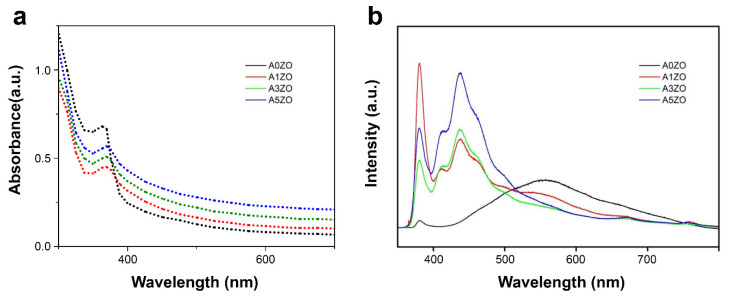
(**a**) UV-Vis DRS absorption spectra in diffuse reflectance spectroscopy and (**b**) photoluminescence spectra of ZnO and Al-doped ZnO nanoparticles at room temperature.

The UV-vis absorption spectra of ZnO and AZO are depicted in Figure 3a. Not only were the edge values of AZO nanoparticles blue-shifted relative to ZnO nanoparticles, but absorbance in the visible region had improved primarily due to deep levels in ZnO’s bandgap created by the increased concentration of defects. The bandgaps of ZnO and AZO, determined with an (α h ν)^2^ plot as a function of photon energy, revealed that higher Al concentrations coincided with lower bandgap energies, which fell from 3.22 to 2.99 eV.

The PL spectra of the pure and Al-doped ZnO nanopowders appear in Figure 3b. The spectrum of the reference sample (i.e., pure ZnO) showed two bands: a narrow one at 380 nm of UV emission and a wide one of visible emission at 552 nm. UV emission was used to refer to band-to-band emissions, while PL emissions in the visible-light range were based on emissions attributed to inter-band defects. As such, the wide visible band was attributed to several intrinsic defects, including interstitial zinc (Zn_i_), oxygen vacancies (V_o_), and oxygen antisites (O_Zn_), present in the ZnO’s structure [28,29].

Beyond that, the PL emission spectra of the Al-doped ZnO samples and pure ZnO samples differed in appearance. As the intensity of the green emission band gradually disappeared, the 380 nm’s emission band increased in intensity. In addition, a new band centered at 437 nm appeared, which we attributed to the blue emission, possibly from intrinsic defects and/or from the recombination of the donor-acceptor pair linked to the acceptor Al. Altogether, our results suggest that, as Al content increased, so did the intensity of the blue band, whereas the green band’s intensity dropped [30].

### 2.2. Photocatalytic Degradation of Cyanotoxins

**Figure 4:**
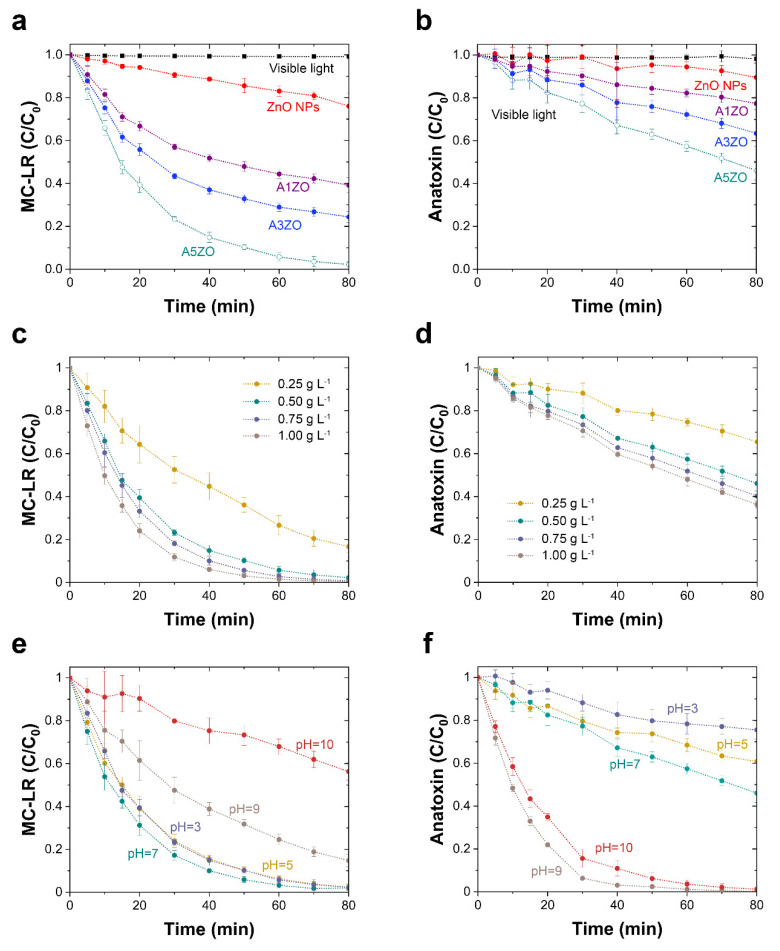
Photocatalytic activity of ZnO and AZO nanoparticle photocatalysts (photocatalyst dosage = 0.5 g L^−1^) in the photocatalytic degradation of (**a**) microcystin-LR (MC-LR) and (**b**) anatoxin-A under visible light-emitting diode (LED) irradiation at pH 7.0 and 20 °C. Effect of A5ZO photocatalyst dosage on the photocatalytic degradation of (**c**) MC-LR and (**d**) anatoxin-A under visible LED irradiation at pH 7.0 and 20 °C. Effect of pH of A5ZO photocatalyst (0.5 g L^−1^) on the photocatalytic degradation of (**e**) MC-LR and (**f**) anatoxin-A under visible LED irradiation at 20 °C. Error bars indicate standard deviations of the four replicated experiments.

The photocatalytic activity of ZnO and AZO nanoparticle photocatalysts was evaluated in terms of the photocatalytic degradation of MC-LR and anatoxin-A under visible-LED irradiation at pH 7.0. As shown in Figure 4a,b, the photolytic degradation of both cyanotoxins was negligible during 80 min of visible-LED irradiation. Prior to investigating the photocatalytic degradation of the cyanotoxins, the time required to attain the adsorption-desorption equilibrium was determined in dark conditions for both the ZnO and AZO nanoparticle photocatalysts. After 60 min, the adsorption-desorption equilibrium was reached for all of the photocatalysts. Thus, only 5 ± 1% and 3 ± 1% of MC-LR and anatoxin-A, respectively, could be adsorbed by ZnO and AZO nanoparticles in dark conditions.

As shown in Figure 4a,b, pure ZnO nanoparticles exhibited the least efficiency in removing MC-LR and anatoxin-A—only approximately 24 ± 3% and 10 ± 2%, respectively—within 80 min. Moreover, as also shown in Figure 4, as Al doping increased, the photocatalytic degradation achieved by AZO nanoparticles with MC-LR and anatoxin-A increased from 61 ± 2% to 98 ± 2% and from 23 ± 2% to 54 ± 1%, respectively, probably due to the improved surface area and enhanced visible-light absorption. Al doping enhanced the photocatalytic performance in the samples, and, for that reason, A5ZO displayed the highest degradation efficiency (i.e., approximately 98 ± 2% and 54 ± 1% for MC-LR and anatoxin-A, respectively) under the same conditions. The effects of the dosage of photocatalyst and the solution’s pH were investigated for the A5ZO nanoparticle photocatalyst only.

The effects the dosage of photocatalyst on the photocatalytic degradation of MC-LR and anatoxin-A under visible LED illumination were systematically assessed by altering the catalyst loading from 0.25 to 1.0 g L^−1^, with all other parameters unchanged. As shown in Figure 4c,d, the photocatalytic degradation of both cyanotoxins increased as the dosage of photocatalyst rose from 0.25 to 0.5 g L^−1^ but less so from 0.5 to 1.0 g L^−1^. In turn, increases in dosage further decreased the degradation rate of both cyanotoxins. In that dynamic, increasing the dosage of the photocatalyst creates more sites for actively adsorbing both cyanotoxins and generates highly reactive radicals, which together translate into an increase in photocatalytic degradation. At the same time, high dosages of photocatalysts (>1.0 g L^−1^) hinder the penetration of visible light and can induce the agglomeration of nanoparticles, which together translate into a reduced rate of photocatalytic degradation. Thus, the optimal dosage for enhanced photocatalytic activity was found to range from 0.5 to 1.0 g L^−1.^

The effect of pH on the photocatalytic degradation of MC-LR and anatoxin-A was assessed in the pH ranges of 3.0 to 10.0 (Figure 4e,f). As is well-known, because the pH of reaction media controls the surface-charge properties of photocatalysts and the speciation of cyanotoxins, it plays a pivotal role in the adsorption and photocatalytic degradation of cyanotoxins. In our study, the isoelectric points of ZnO and A5ZO were identified to be pH 8.8 and pH 9.0, respectively. Such findings suggest that, when the pH is less than the isoelectric point, the surface is positively charged; when the pH equals the isoelectric point, the surface is neutral; and when the pH is greater than the isoelectric point, the surface is negatively charged. By comparison, MC-LR was positively charged when the pH was less than 2.09 neutral when the pH ranged between 2.09 and 2.19, and negatively charged when the pH was higher than 2.19 [9]. However, anatoxin-A, the dominant species of this cyanotoxin, was neutral at a pH less than 9.36 [9].

As shown in Figure 4e, the photocatalytic degradation of MC-LR was higher and virtually identical after 80 min of visible-LED irradiation at pH 3.0 to 7.0, when the photocatalyst surface was positively charged and the dominant species of MC-LR in the reactant media was negatively charged. As expected, further increases in pH translated into less photocatalytic degradation when both the photocatalyst’s surface and the cyanotoxins were negatively charged, which promoted the electrostatic repulsion, while hindering the interaction between both entities. By contrast, the maximum photocatalytic degradation of anatoxin-A occurred at pH 9.0 (i.e., 99.8 ± 0.2%) to 10.0 (i.e., 99.6 ± 0.4%), when A5ZO was negatively charged and anatoxin-A was neutral (Figure 4f). At a pH lower than 9.0, because both A5ZO and anatoxin-A were negatively charged, the interaction between the entities was unfavorable, which effectively reduced the efficiency of photocatalytic degradation. Taken together, it is well-known that acidic media are not optimal for ZnO, because they affect the photocatalyst’s stability as a result of ZnO’s dissolution. However, in the presence of cyanobacterial blooms, the pH of bodies of water increases and can exceed 8.0, due to the consumption of dissolved CO_2_. Thus, the photocatalytic degradation of cyanotoxins is necessary and has to be efficient at pH levels close to neutral or slightly alkaline [9].

**Figure 5:**
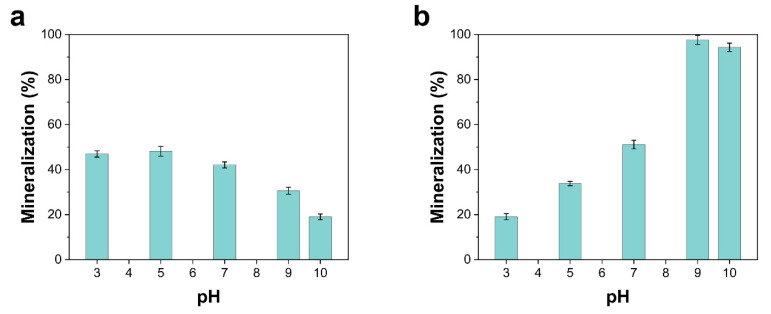
Mineralization (180 min) as a function of pH for AZO nanoparticle photocatalysts (photocatalyst dosage = 0.5 g L^−1^) of (**a**) microcystin-LR and (**b**) anatoxin-A under visible-light-emitting-diode irradiation at 20 °C. Error bars indicate standard deviations of the four replicated experiments.

The aim of treating water photocatalytically is to completely mineralize pollutants—in our case, cyanotoxins. The photocatalytic degradation of MC-LR and anatoxin-A produces numerous organic intermediates as they mineralize [9,31,32,33,34]. As shown in Figure 5, the mineralization of MC-LR after 180 min of visible-LED irradiation was less than 50% of the theoretical amount expected for complete mineralization at pH ≤ 7.0. In the case of anatoxin-A, practically complete mineralization occurred at pH ≥ 9.0 after 180 min of visible-LED irradiation. Such results imply that the mechanism of MC-LR’s degradation is more complex than the one proposed for anatoxin-A. The complete mineralization of both cyanotoxins was confirmed after 380 min of visible-LED irradiation. At the same time, the reported values are highly competitive with outcomes achieved with state-of-the-art photocatalytic techniques for mineralizing cyanotoxins [9,12,13,31,35,36,37]. By contrast, most studies on the photocatalytic degradation of cyanotoxins have not involved analyzing mineralization but only the degradation of the initial cyanotoxins, often without considering that some by-products and/or intermediates could be as harmful as the cyanotoxins [9,12,13,31,35,36,37].

It is significant that the cost associated with the photocatalytic treatment of cyanotoxins (or organic pollutants in general) is typically translated directly to the energy consumption, which is closely related to the wattage of the lamp. Therefore, the use of visible-LED irradiation should reduce the energy consumption versus conventional light sources. However, large-scale experiments, incorporation of photocatalysts in reactors, photocatalytic reactor design, and water-matrix effects, among others, must be necessarily considered to predict costs [9,38,39,40,41].

### 2.3. Photocatalyst Reusability

**Figure 6:**
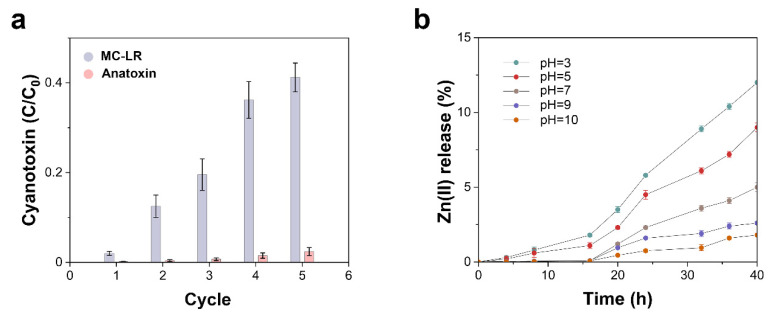
(**a**) Degradation of MC-LR at pH 7.0 and anatoxin-A at pH 9.0, in five consecutive reusability experiments with A5ZO nanoparticle photocatalysts (photocatalyst dosage = 0.5 g L^−1^), after 80 min of visible light-emitting diode (LED) irradiation at 20 °C. (**b**) Time-dependent concentration of Zn(II) ions from A5ZO at different pH levels during 40 h of visible-LED irradiation at 20 °C. Error bars indicate standard deviations of the four replicated experiments.

The reusability and stability of A5ZO nanoparticles in the degradation of MC-LR at pH 7.0 and anatoxin-A at pH 9.0 were also investigated. Figure 6a shows the five consecutive cyclic photocatalytic degradations of both cyanotoxins after 80 min irradiation under visible LED. For MC-LR, the efficiency of photocatalytic degradation dropped significantly after five cycles, possibly due to the adsorption of by-products formed during MC-LR’s degradation, which results in photocatalyst poisoning. At pH 7.0, MC-LR’s mineralization after 80 min of irradiation was less than 20 ± 2%. Conversely, the photocatalytic degradation of anatoxin-A was virtually constant during the 80 min of five consecutive cycles of irradiation, as well as especially efficient (i.e., 98 ± 2%) with A5ZO. By contrast, at pH 9.0, the mineralization of anatoxin-A after 80 min of visible-LED irradiation was approximately 67 ± 2%. The different mineralization rate of MC-LR at pH 7.0 and anatoxin-A at pH 9.0 seemed to affect the reusability of A5ZO, which was higher when the rate of mineralization was greater.

The chemical and photochemical stability of A5ZO was also investigated by analyzing the Zn(II) and Al(III) released in aqueous media at different pH levels for 40 h of visible-LED irradiation. ZnO’s well-known photocorrosive behavior occurs under UV and visible-light irradiation, and at extreme pH values (i.e., 2.0 < pH > 10.0), ZnO even dissolves, which of course defeats its purpose in photocatalysis [39]. However, corrosion and photocorrosion can be suppressed via surface modification or doping strategies. As shown in Figure 6b, the corrosion and photocorrosion of A5ZO nanoparticles were minimal in weak alkaline conditions and moderate at neutral pH. Importantly, no Al(III) species in the aqueous medium were detected during the 40 h of visible-LED irradiation. Such findings matter because cyanotoxins are usually generated in neutral or slightly alkaline bodies of water. The findings also confirm the stability of synthesized A5ZO photocatalysts in the photocatalytic degradation of cyanotoxins from natural bodies of water.

## 3. Conclusions

In search of a complementary economic gain relative to energy consumption, we examined the photocatalytic activity of Al-doped ZnO nanoparticles in degrading and mineralizing two cyanotoxins under visible-LED irradiation. The sol-gel method was found to be as reproducible method of synthetizing well-defined Al-doped ZnO nanoparticles with controlled percentages of Al, namely ranging from 0 to 5 at.%. Doping has a twofold effect: It reducing bandgaps, thus widening the visible activity; and it minimizes deleterious photocorrosion, that is, the easy photochemical dissolution of zinc oxide.

Increasing the dosage of photocatalyst in the solution did not exert a linear effect on the photocatalytic activity. Upon having a certain amount of photocatalyst, photocatalytic activity increased; however, similar behavior was observed from a threshold value as well (0.5 g L^−1^). Considering that the easy agglomeration of the nanoparticles could have been responsible, we pragmatically decided that a dose of 0.5 g L^−1^ could maximize efficacy and save material.

Among other findings, the pH of the solution for mineralizing cyanotoxins was critical. The optimal pH is the one that favors the interaction of the substrate and the photocatalyst, which itself relates to the surface charge of the species. Because the pH value is responsible for the molecule’s chemical form, and thus its electrical charge, the value of the isoelectric point of the species proved critical as well. According to our results, the best pH value for solutions for mineralizing anatoxin-A is 9.0, under which conditions mineralization completed, and for MC-LR is pH 7.0, under which conditions the reaction yield became balanced with the photocatalyst’s chemical stability. The reusability of the photocatalysts also depended on the entities and reaction conditions. In any case, neutral or weak alkaline media are best suited to avoiding the risk of the photocorrosion or dissolution of the photocatalyst, as has been proven by controlling the release of Zn(II).

The results additionally show that the photocatalytic activity of the synthesized photocatalysts was greater than that previously reported for degradation of cyanotoxins. Even when mineralization remains incomplete, as was the case with MC-LR, degrading a high percentage of any pollutant benefits the environment, because the intermediate-reaction products exert lower harmful effects on water.

## 4. Materials and Methods

### 4.1. Synthesis of Nanoparticles

**Scheme 1:**
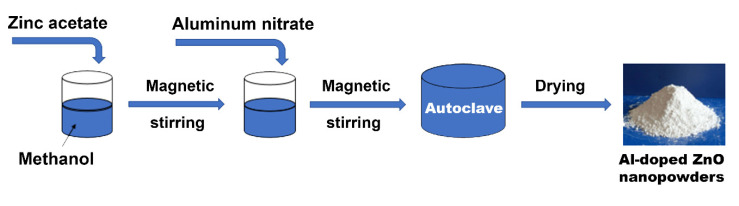
Synthesis of AZO nanoparticles by the sol-gel method.

Al-doped ZnO nanopowders were elaborated via the sol–gel method, as shown in Scheme 1. We dissolved 16 g of zinc acetate dihydrate (Zn(CH_3_COO)_2_·2H_2_O; ≥99.0%, Sigma-Aldrich) in 112 mL of methanol (99.8%, Sigma-Aldrich), which acted as solvent. The solution was stirred for 10 min, at room temperature, after which we added an adequate quantity of aluminum nitrate-9-hydrate (≥98%, Sigma-Aldrich) as a dopant precursor, according to Al:Zn atomic ratios of 0%, 1%, 3%, and 5%. The obtained solutions were stirred for 15 min and placed in an autoclave, to dry in the supercritical condition of ethyl alcohol. Last, the synthesized powders were calcined for 2 h, using an air muffle furnace at 400 °C.

### 4.2. Materials Characterization

The surface morphology, crystalline structure, and optical properties of ZnO-based photocatalysts were investigated by TEM, XRD, and PL measurements. First, we studied the microstructure of the samples by using an XRD machine (Bruker AXS D8 Advance, MA, USA), at the wavelength of *CuKα* (i.e., 1.5405 Å). The network parameters (*a*,*c*) and the unit cell volume (*V*) were determined by using the following equations [23,24]:

(1) $$a=\frac{\lambda }{\sqrt{3}\sin\theta_{\left(100\right)}}$$

(2) $$c=\frac{\lambda }{\sin\theta_{\left(002\right)}}$$

(3) $$V=\frac{\sqrt{3}}{2}a^{2}\cdot c$$

where *λ* is the wavelength of the radiation used (i.e., 1.54060 Å for *CuKα*) and *θ* is the Bragg diffraction angle. The calculated lattice parameters values (*a,c*) and the concluded unit cell volume (*V*) are presented in Table 1. The micro-deformations (*ε*) were estimated according to the Williamson-Hall model [25]:

(4) $$\beta \cos\theta =\frac{k\lambda }{D}+4\;\varepsilon \sin\theta$$

The plot of (*β*cos*θ*) versus (4sin*θ*) for the ZnO nanopowder, as shown in Figure 1, was fitted according to Equation (4). The *D* value was estimated from the slope of the extrapolation of the linear fit, while the ε value was given by the fit slope. In addition, the dislocation density (*δ*) was calculated by using the following relationship [26]:

(5) $$\delta =\frac{15\;\varepsilon }{a\;D}$$

Next, the shapes and sizes of the prepared nanoparticles were examined with a transmission electron microscope (JEOL JEM 2010, Tokyo, Japan), an LaB6 electron gun that operates at 200, which was equipped with a Gatan 794 MultiScan CCD camera used to view the digital images. The Brunauer-Emmett-Teller (BET) surface area of each photocatalyst was determined by using the N_2_ adsorption-desorption isotherms obtained at 77 K, using a Micrometrics Tristar-II (Micrometrics, Canada). The UV-vis diffuse reflectance spectra (DRS) were recorded by using a Lambda 900 UV spectrophotometer (PerkinElmer, Waltham, MA, USA). Last, a NanoLog Horiba modular spectrofluorometer (Horiba, Kyoto, Japan) used for PL measurements was equipped with an Xe lamp, as the excitation light source, with a wavelength of 325 nm, at room temperature. Emissions were studied between 350 and 800 nm.

### 4.3. Photocatalytic Activity

To examine the photocatalytic activity of the Al-doped ZnO nanoparticles, the photocatalytic degradation of single-pollutant aqueous solutions of 1.25 mg L^−1^ MC-LR (Sigma-Aldrich) and 2.5 mg L^−1^ (±)-anatoxin-A fumarate (Hello Bio Ltd., >99%) was observed under irradiation from three 6.2 W LEDs (irradiance = 65 mW cm^−2^; energy consumption = 7 kWh or 1000 h) for 80 min. The photocatalytic experiments were conducted in a quartz dish reactor (volume = 40 mL, diameter = 10 cm) sealed with a quartz cover and cooled with fans, to prevent the evaporation of the solution. All the experiments were performed in quadruplicate. Different dosages of Al-doped ZnO nanoparticles (i.e., 0.25, 0.5, 0.75, and 1.0 g L^−1^) were ultrasonically (power = 5 W L^−1^; time = 60 s) suspended in the pollutant solution, to determine the effect of the dosage of the photocatalyst. To examine the effect of pH on the photocatalytic degradation of the cyanotoxins, the initial solution’s pH was adjusted to 3.0, 5.0, 7.0, 9.0, and 10.0 with 0.1 M HCl or NaOH solutions. Before irradiation commenced, the suspensions were magnetically stirred at 200 rpm, in dark conditions, in the presence of each cyanotoxin, for 1 h, in order to achieve adsorption-desorption equilibrium. Once achieved, the suspensions were irradiated with visible light by way of continuous magnetic stirring. With the temperature maintained at 20 °C, samples (500 µL) were taken at specified times and filtered through a syringe filter (0.02 μm, Whatman), to separate the photocatalyst. The temporal photocatalytic degradation of cyanotoxins was monitored by quantifying the reduction of the concentration of MC-LR and anatoxin-A, using a high-performance liquid chromatograph (Agilent; Santa Clara, CA, USA) with a photodiode array detector set at 238 nm and ultrahigh-performance liquid chromatography (Thermo Fisher Scientific; Waltham, MA, USA), respectively.

To quantify MC-LR, a C18 Discovery HS column 150 mm by 2.1 mm i.d. for particles 5 μm in size (Supelco, Inc.; Bellefonte, PA, USA) was used for the stationary and mobile phases; it consisted of 0.05% (*v/v*) trifluoroacetic acid in Milli-Q water and 0.05% trifluoroacetic acid in acetonitrile in a ratio of 60:40. Analysis was performed under equilibrium conditions at a column temperature of 40 °C, a flow rate of 0.2 mL min^−1^, and an injection volume of 20 μL [40]. By contrast, to quantify anatoxin-A, a Kinetex HILIC column measuring 100 mm by 2.1 mm i.d. for particles 2.6 μm in size (Phenomenex, Inc.; Basel, Switzerland) was employed for the stationary phase, while the mobile phase consisted of 95% (*v/v*) acetonitrile +5% (*v/v*) aqueous solution (i.e., 2 mM ammonium formate +3.6 mM formic acid) and an aqueous solution of 2 mM ammonium formate and 3.6 mM formic acid in a ratio of 85:15 [12]. The analysis was performed under equilibrium conditions, with a column temperature of 25 °C, a flow rate of 0.2 mL min^−1^, and an injection volume of 20 μL. Last, the mineralization of cyanotoxins was determined by comparing the reduction of total organic content (TOC) after 180 min of visible LED irradiation, using a TOC analyzer (Shimadzu; Nakagyo-ku, Kyoto, Japan). The photocatalytic experiments for each catalyst were performed in quadruplicate.

### 4.4. Photocatalyst Reusability

To evaluate the reusability of the Al-doped ZnO nanoparticles, the photocatalytic degradation of MC-LR (Sigma-Aldrich) and (±)-anatoxin-A fumarate was analyzed by reusing the same photocatalyst sample, under the same reaction conditions, during five consecutive 80 min irradiation cycles. For comparison, the photocatalytic degradation of cyanotoxins was also determined by liquid chromatography after 80 min of visible LED irradiation. After each experiment, the Al-doped nanoparticles were recuperated by centrifugation, washed, and reused. Meanwhile, photostability was analyzed by quantifying the time-dependent evolution concentration of Zn(II) ions in 40 mL Milli-Q water, under visible LED irradiation for 8 h. The initial solution’s pH was adjusted to 3.0, 5.0, 7.0, 9.0, and 10.0 with 0.1 M HCl or NaOH solutions. At different times during the 40 h period, 0.5 mL of irradiated solution was filtered through a syringe filter (0.02 μm, Whatman), to separate the photocatalyst. The concentration of Zn(II) ions was determined spectrophotometrically by using Zincon monosodium salt (Sigma-Aldrich) in borate buffer (50 mM, pH = 9) and measuring the absorbance associated with the Zn(II)-bound Zincon complex at 620 nm [42,43]. For those measurements, a UV-1800 Shimadzu UV-vis spectrophotometer (Shimadzu; Japan), accompanied by a quartz cuvette with an optical length of 1 cm, was used. The Al(III) concentration was determined by ICP–OES analysis. The dosage of the photocatalyst for all experiments described in this section was set at 0.5 g L^−1^. The reusability experiments were performed in quadruplicate.

## Figures and Tables

**Table 1:** Structural parameters of the AZO samples.

	*a* (Å)	*c* (Å)	*V* (Å^3^)	*D_W-H_* (nm)	*D_TEM_* (nm)	*ε* (10^−4^)	*δ* (10^−3^ Line × nm^−2^)
A0ZO	3.244	5.195	47.338	52	39	9.1	81
A1ZO	3.246	5.201	47.465	32	33	12.0	174
A3ZO	3.245	5.200	47.431	66	43	16.5	116
A5ZO	3.245	5.199	47.423	69	48	22.2	148

## Data Availability

Data is contained within the article.
